# Minimizing complications in bikini incision direct anterior approach total hip arthroplasty: A single surgeon series of 865 cases

**DOI:** 10.1186/s40634-020-00318-7

**Published:** 2021-01-04

**Authors:** Avinash Alva, Ikram Nizam, Sophia Gogos

**Affiliations:** 1 Mulgrave Private Hospital, Cnr Police Rd and Gladstone Rd, Mulgrave, VIC 3170 Australia; 2 Centre for Adult Joint Arthroplasty, 1356 High Street, Malvern, VIC 3144 Australia; 3 Alfred Hospital, 55 Commercial Road, Melbourne, VIC 3004 Australia

**Keywords:** Bikini incision, Total hip arthroplasty, Total hip replacement, Direct anterior approach, Complications, Enhanced recovery

## Abstract

**Purpose:**

The purpose of this study was to report all complications during the first consecutive 865 cases of bikini incision direct anterior approach (DAA) total hip arthroplasty (THA) performed by a single surgeon. The secondary aims of the study are to report our clinical outcomes and implant survivorship. We discuss our surgical technique to minimize complication rates during the procedure.

**Methods:**

We undertook a retrospective analysis of our complications, clinical outcomes and implant survivorship of 865 DAA THA’s over a period of 6 years (mean = 3.9yrs from 0.9 to 6.8 years).

**Results:**

The complication rates identified in this study were low. Medium term survival at minimum 2-year survival and revision as the end point, was 99.53% and 99.84% for the stem and acetabular components respectively. Womac score improved from 49 (range 40–58) preoperatively to 3.5(range 0–8.8) and similarly, HHS scores improved from 53(range 40–56) to 92.5(range 63–100) at final follow-up (mean = 3.9 yrs) when compared to preoperative scores.

**Conclusions:**

These results suggest that bikini incision DAA technique can be safely utilised to perform THA.

## Introduction

Rapid functional recovery through minimally invasive techniques for THA has received increased attention in recent times [1, 18]. Recent studies indicate that the advantages of DAA include tissue preservation, early functional recovery, decreased post-operative pain, increased accuracy of implant alignment, decreased risk of dislocation, lower risk of revision for instability, smaller leg length discrepancies and improved patient satisfaction at one year following DAA compared to posterior approach [5, 39]. The DAA to the hip using a cosmetic transverse bikini incision allows access to the hip in a true inter-nervous plane, thus minimising damage to the abductor mechanism and complications of the longitudinal incision [4, 35, 36]. Many studies have reported a high complication rate with DAA and attributed this to a learning curve [7, 10, 12, 40, 49]. With appropriate training and precautions, DAA THA may be safely performed with similar complication rates when compared to the posterior approach [43]. The primary aim of this study was to report all complications during bikini incision DAA THA performed by a single surgeon using a standard operating table. The secondary aims are to report clinical outcomes and implant survivorship. The complication rates and technical steps undertaken to avoid common complications are discussed Table 1.

**Table 1:** Overview of complications found in our series (851 hips with average 3.9 years follow-up) and results from published studies on DAA and bikini incision DAA THA

Complication	Results in current study (%)	Results from published literature on DAA and Bikini incision DAA THA (references)
Superficial wound infection	3 (0.35)	0.3–4% [21, 32, 50, 54]
Deep wound infection	2 (0.23)	0.3–0.8% [20, 29, 33]
Dislocation	2 (0.23)	0.3–2.7% [11, 20, 35]
Calcar fracture	3 (0.35)	0.3–1.5% [20, 35, 40]
Stem subsidence	3 (0.35)	0.2–0.7% [35, 40]
Trochanteric fracture	3 (0.35)	2.3% [29]
Femoral fracture	4 (0.47)	0.12%-0.45% [19, 29]
Leg length discrepancy	2 (0.23)	0.2% [35]
Deep vein thrombosis	3 (0.35)	0.8–1.35% [11, 30]
Permanent neuropraxia (LFCN)	3 (0.35)	1.2–11% [25, 33, 35]
Transient neuropraxia (LFCN)	55 (6.4)	15–81% [17, 20, 45]
Canal perforation	0	0.8–9% [16, 52]
Trochanteric Bursitis	8 (1.1)	6.1% [48]

## Materials and methods

**Fig. 1:**
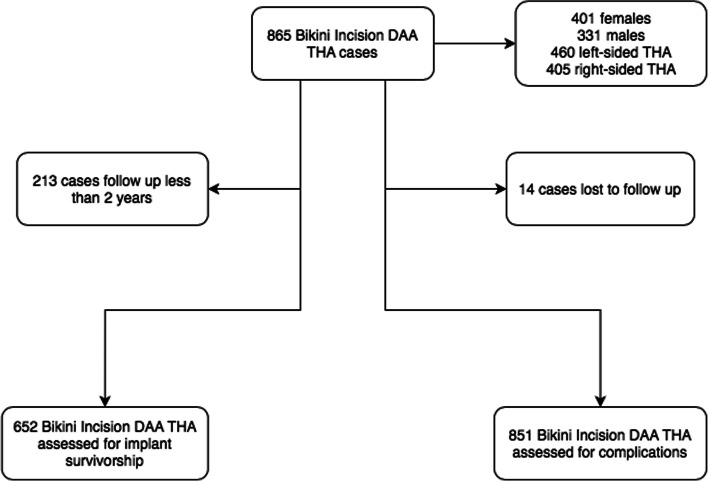
Flow chart with overview of study

A retrospective analysis of elective primary total hip arthroplasties performed by a single surgeon on a standard operating table between May 2013 and December 2019 (Fig. 1) were included in the study. All procedures were performed utilizing the bikini incision DAA regardless of the indication for surgery or body mass index. Patient demographics, indications for surgery and patient reported outcome measures were prospectively recorded. Complications were documented in operative notes and subsequent consultation notes throughout post-operative follow-up. Patients who underwent posterior total hip replacement (indicated in the presence of intestinal stoma, those with concomitant abductor tendon tears, with a fractured neck of femur) were excluded from the study. Institutional review board approval was obtained for this study as appropriate.

The modified vessel sparing bikini anterior approach, namely a horizontal incision over the lateral groin crease, was utilized in all cases [35, 42]. A superolateral capsular window was utilised preserving the ascending branches of the circumflex femoral vessels. A dedicated fracture table or intraoperative radiography was not routinely used in any of the cases. All procedures were performed by the senior author who was fellowship trained in direct anterior approach hip arthroplasty. Rapid recovery protocols were instituted for all cases [31].

Patient reported outcome measures were prospectively collected both pre- and post-operatively using Harris Hip scores (HHS) and The Western Ontario and McMaster Universities Osteoarthritis Index (Womac) scores. Intra-operative and post-operative complications were identified from patient records and plain radiographs. Antero-posterior pelvic x-rays were routinely obtained day one post-operatively, six months post-operatively and annually thereafter. Post-operative x-rays were assessed for fractures, dislocations, implant loosening, stem subsidence, heterotrophic ossification and leg length discrepancy. Measurements were performed on digital radiographs (Intelerad Medical Systems Incorporated, Quebec, Canada) to identify stem subsidence. Subsidence was measured as the perpendicular distance from the trochanter to the shoulder of the stem [9]. Leg length discrepancy was measured on AP Pelvis x-ray as the distance from the line at the inferior aspect of the ischial tuberosities to the most prominent medial point of the lesser trochanter [23]. Implant survival was estimated using Kaplan Meyer survival analysis using implant revision for any reason as the endpoint. Implant survivorship was calculated for cases with a minimum follow up of 2 years. Since there is significant overlap between complications of DAA and bikini incision DAA, a literature search was performed on open athens to identify complication rates reported during DAA THA and bikini DAA approach. We utilised the literature search to discuss our complication rates.

### Clinical protocols and surgical technique

All patients were provided with a detailed booklet pre-operatively with information regarding the peri-operative period and postoperative expectations, as reports suggested mental preparation can impact early recovery [28]. Since 2018, the author recommends the patient commence oral supplements (Zinc, selenium, iron, Vitamin C, Vitamin D) two weeks pre-operatively to optimise their nutritional status [3]. Patients were advised to bathe using an antibacterial skin emulsion daily for upto 7 days before surgery [6]. Patients over the age of 75 years and those with premorbid medical conditions were reviewed by a perioperative physician before surgery.

A traction table or leg holders were not used intra-operatively in any of the cases reported. The standard operating table included a table break at the level of the hip joint to ensure hip extension and provide adequate access and delivery of the proximal femur for broaching. A 6–8 cm skin incision was made in the lateral groin crease, starting inferior to the ASIS and extending laterally. This approach utilises the subfascial plane between the tensor fascia latae [TFL] and sartorius, preserving the branches of the lateral circumflex femoral vessels and the anteromedial capsule [35, 42].

**Fig. 2:**
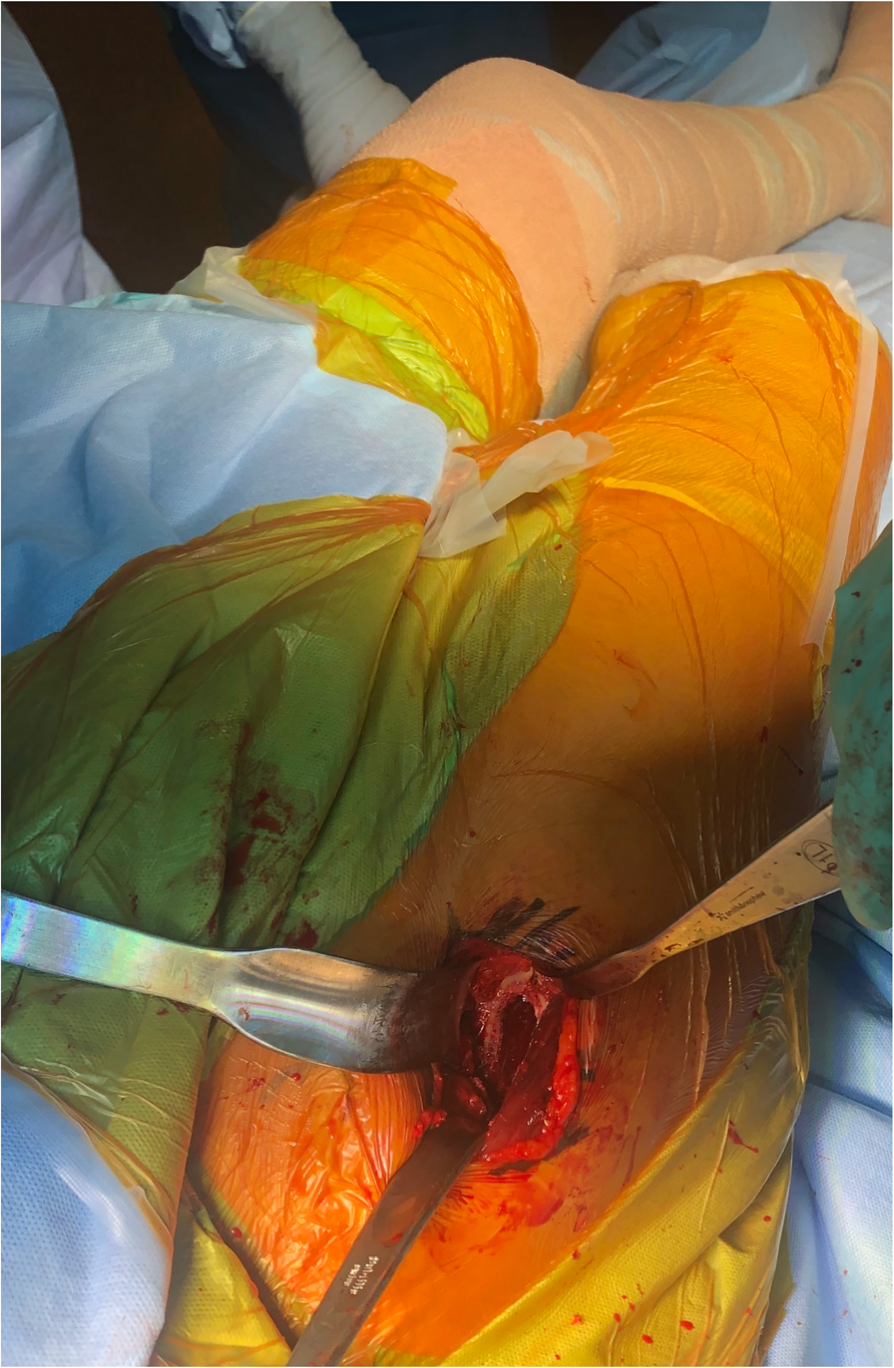
Surgeon’s view of the proximal femur during a bikini DAA THA with the table-break providing hip extension

A capsular incision exposes the femoral neck. After excising the head and neck, a secondary capsular release is performed with the leg in a figure of four position stretching the postero-superior capsule. The femur was first prepared with the operative leg placed in a figure of lazy four position under the contralateral leg, the table broken to 30–45 degrees and the broach left in situ (Fig. 2). A rat tailed rasp customised with increased curve is used to find the canal. The surgeon routinely used the Woodpecker pneumatic broaching system (IMT USA, LLC) to prepare the femur. The acetabulum was then prepared with the table levelled. Intra-operative imaging was not used in any of the procedures and two screws were placed through the acetabular shell in majority of cases. Trial reductions were performed for stability and leg lengths were measured using the two medial malleoli with the pelvis in neutral position.

Oxinium (Smith and Nephew Memphis, TN) femoral heads were used in all cases, with the femoral head size determined by cup size. 48 mm acetabular shells (Acetabular shell: R3 three hole HA coated Smith and Nephew Memphis, TN) were used for femoral heads less than or equal to 32 mm in diameter. Larger femoral heads (> 36 mm) were encased in 52 mm shells and above. A highly cross-linked R3 polyethylene liner was used in all cases with Polar cementless femoral stem (Smith and Nephew AG, Baar, Switzerland) and CPCS cemented stems (CPCS Smith and Nephew, Memphis TN). Cemented femoral components were preferentially used in patients over the age of 75 years, bone density test T-score of less than or equal to -2.5 (diagnostic of osteoporosis), Dorr type C femurs, currently taking steroids or anticoagulants or where poor bone quality was suspected by the operating surgeon during femoral broaching. Fourth generation cementation techniques were used including vacuum mixing system, pulsatile lavage, cement guns, cement restrictor, distal stem centraliser and sustained pressurisation. Simplex HV with Gentamycin cement was used (Stryker Howmedica Osteonics, USA).

Perioperative intravenous cefuroxime was routinely used on induction. Local infiltrative anaesthesia (3–4 doses of bupivacaine 0.5% 20 ml every 6 h) via an intra-articular catheter was administered for 24 h postoperatively routinely [31]. Skin closure was achieved using Monocryl monofilament absorbable sutures and a thin Comfeel (Coloplast Pty Ltd, Victoria, Australia) pressure dressing applied.

All patients underwent post-operative rehabilitation protocol with early mobilisation and discharge [31]. Postoperatively, patients were cautioned against combined hip extension and external rotation. Patients were encouraged to mobilise within 3 to 4 h of the procedure, allowing for cessation of the spinal anaesthetic. Mechanical thrombo-prophylaxis in the form of above knee thromboembolic deterrent stockings and pneumatic calf compressors were used in conjunction with chemical prophylaxis (aspirin 300 oral daily therapy unless on oral anticoagulants preoperatively) in all patients.

## Results

Over the 6-year study period a total of 865 bikini THAs were performed on 732 patients, including nine cases performed as part of bilateral THA. There were 331 males, 401 females, 460 left and 405 right hips. Average age at the time of operation was 69 (range 32–91). The average duration of follow-up was 0.9 to 6.8 years (mean = 3.9yrs). The average BMI at the time of surgery was 28 (range 18–44). A total of 223 cemented CPCS femoral stems and 642 polar cementless stems were used. Indication for surgery included primary osteoarthritis (77%), developmental dysplasia of the hip (16%), rheumatoid arthritis (2.5%), sequelae of Perthes disease (1.5%) and post-traumatic arthritis (3%). The average length of surgery was 71 min (45–98 min) and mean duration of hospital stay was 1.6 days (range 1–4 days).

We noted the following complications in this series. We identified neuropraxia of the lateral femoral cutaneous nerve of the thigh (LFCN) in 55 patients (6.4%) who reported temporary hypoesthesia that resolved within two years and three patients (0.35%) reporting permanent hypoesthesia. Three patients (0.35%) had a trochanteric fracture and four patients (0.47%) had a femoral fracture. Post-operative infections included three (0.35%) superficial wound infections and two (0.23%) incidences of deep infections. There were two cases (0.23%) of hip dislocation, three cases (0.35%) of stem subsidence and three (0.35%) calcar fractures. Post-operatively, two patients (0.23%) had a leg length discrepancy and three patients (0.35%) suffered a deep vein thrombosis. Eight patients (1.1%) suffered from ipsilateral trochanteric bursitis post-operatively. There were 3 patients (0.35%) who developed postoperative iliopsoas tendinitis. No canal perforations or heterotropic ossifications were reported in this study.

The average HHS improved from 53 (range 40–56) pre-operatively to 92.5 (range 63–100) post-operatively at final follow -up. Similarly, average Womac scores improved from 49 (range 40–58) pre-operatively to 3.5 (range 0–8.8) at final follow-up. 213 cases had a follow-up of less than 2 years. Of the 652 cases with over 2 years follow-up one cup was revised for infection and 3 stems were revised. Using revision of implant as endpoint at final follow-up and minimum of 2 years follow-up, mean survival of stem and cup were 99.53% and 99.84% respectively. 14 cases were lost to follow-up.

## Discussion

Understandably, there is a degree of concern during inception and learning of novel surgical techniques such as DAA THR, with presumed implications on the incidence of complications [22, 55]. Previously reported complications of DAA include lateral femoral cutaneous nerve (LFCN) dysfunction, intra-operative fractures, early revision for component loosening, femoral nerve palsy, superficial and deep infections, leg length discrepancy, excessive bleeding and heterotopic ossification [33].

### Neuropraxia

Injury to the lateral femoral cutaneous nerve (LFCN) has been previously reported as a potential complication of DAA [33, 39]. In this series, 58(6.75%) patients were diagnosed with post-operative neuropraxia, 55(94.8%) of which completely resolved within two years post-operatively. None of these patients reported functional problems secondary to this painless sensory deficit. The incidence of both temporary and permanent neuropraxia affecting the LFCN varies widely in reported literature. The rate of transient LFCN neuropraxia reported in this study (6.4%) was higher than comparable studies (1.5%), although the significance of this result in the context of varying data is unclear [20]. Recent reports suggest that the bikini incision may be protective for the LFCN [14]. To minimise the risk of LFCN injury, we recommend that the TFL fascia is carefully incised lateral to the intermuscular plane during surgical exposure, before commencing deep dissection in the subfascial plane.

### Fractures

**Fig. 3:**
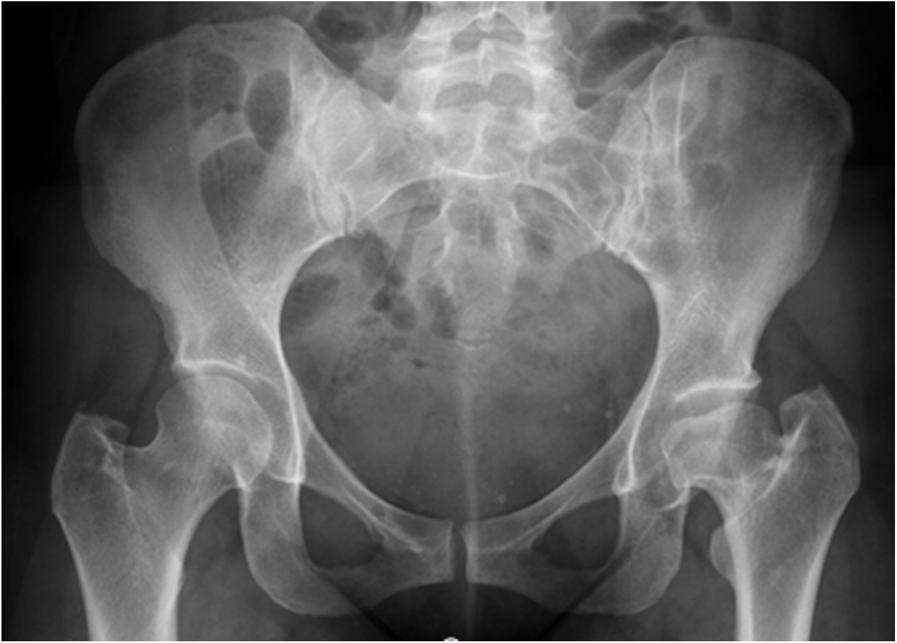
X-ray of a dysplastic left hip with short neck and flattened head

**Fig. 4:**
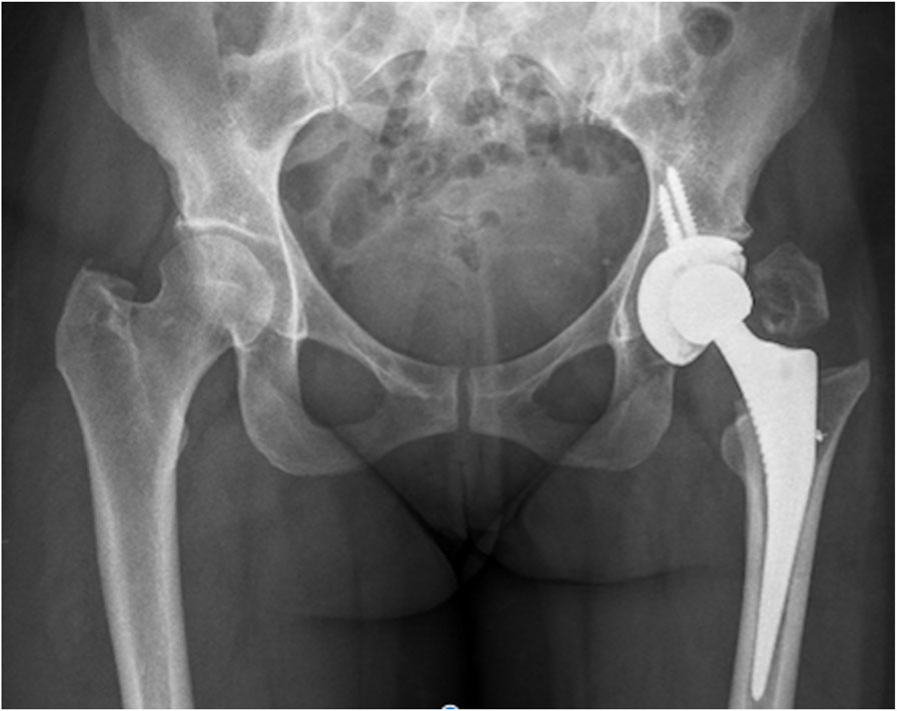
X-ray of postoperative displacement of trochanteric fracture in the case of dysplastic hip

**Fig. 5:**
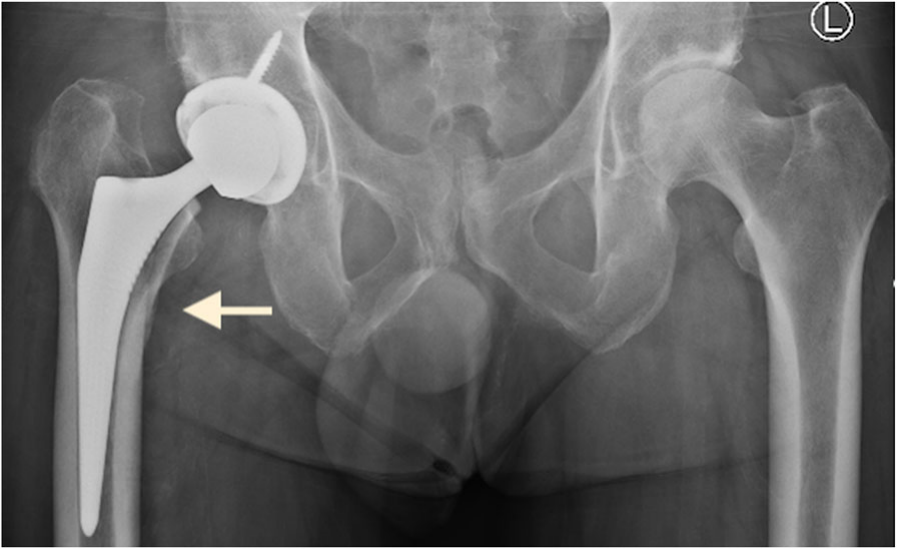
X-ray of femoral fracture with subsidence of stem

Fractures in this series included three trochanteric fractures (0.35%), four femoral shaft fractures (0.47%) (one intraoperative and three postoperative) and three intraoperative calcar fractures (0.35%). A meta-analysis of adverse effects of DAA THA by De Geest et al. reported an incidence of femoral fractures of 0.5% and trochanteric fractures of 0.8% [11]. The trochanteric fractures in our series were noted in cases with a short neck or flattened dysplastic head (Fig. 3 and 4), which led to inadvertent fractures during resection of the head/neck or excessive levering with femoral retractor. Sang et al. have reported the impact of hip anatomy (low distance between greater trochanter and anterior superior iliac spine) on complication rates in DAA THA [46]. Two trochanter fractures were managed with trochanteric plate and one undisplaced fracture was treated successfully with protected weight bearing for 6 weeks. Calcar fractures were treated with circlage wiring and protected weight bearing for 6 weeks. All four femoral shaft fractures (Fig. 5) were treated with cable or plate fixation with one case requiring stem revision. We recommend that adequate femoral elevation and exposure is achieved to provide the appropriate trajectory for femoral broaching. Using a canal finder and clearing the lateral cortex minimises varus positioning and risk of lateral cortical perforation. Since the lead surgeon in our series had crossed the learning curve prior to this study, we did not utilize x-rays routinely during surgery. We however recommend the use of x-rays during the learning curve to achieve appropriate femoral stem sizes and acetabular component orientation.

### Stem subsidence and early femoral failure

Leunig et al. reported an incidence of 0.2% stem subsidence post-operatively, a slight decrease compared to the three cases of stem subsidence (0.35%) reported in this study [35]. A commonly reported challenge in DAA is poor femoral exposure leading to under-sizing, implant subsidence and early femoral failure [38]. Femoral complications can be minimised with precise preoperative templating, sequential soft tissue releases, methodical exposure and delivery of the proximal femur. Capsular releases enable external rotation of the femur, sufficient to safely work on the femur without damaging the abductors. The femur may be elevated with a bone hook to stretch the tissues and improve femoral exposure.

### Dislocation

Two patients (0.23%) had posterior joint dislocations post-operatively in this series. A dislocation rate of 0.6% was reported by Leunig et al. who utilized the bikini incision DAA [35]. In our series, one dislocation occurred as a dashboard injury in a car crash and the other was recorded 3 weeks post operatively after a fall. Preservation of the posteromedial capsule and short external rotators contributed to a low dislocation rate in our series. A customized cup alignment guide was used to seat the cup in the desired inclination and anteversion. An anatomical bone-preserving cup position as opposed to the traditional medialization was utilised in majority of the cases [2].

### Cortical perforation

We did not come across any cases of lateral cortical perforation. Perforation is a known complication of DAA due to incorrect trajectory of femoral broaching [16]. We routinely try to ensure that the stem position is suitably lateralized, minimising the likelihood of lateral stem perforation. The use of X-rays during the step may be particularly useful for surgeons during their learning curve.

### Traction table complications

No traction table or leg holders were used in any case during this series. DAA THA undertaken with a standard table has shown to provide short-term perioperative benefits like decreased blood loss, lower incidence of intraoperative fractures and shorter operative time [47].

### Infection

An increased incidence of superficial wound dehiscence and deep infections has been reported with DAA THR [7]. The incidence of deep wound infection reported in this study is lower than the 0.88% reported by Jewett and Collis in their series of DAA THA [29]. Low infection rate in our series may be attributed to reduced operation times, careful haemostasis and soft tissue retraction. In the majority of cases in this series, the lateral femoral circumflex vessels were preserved to reduce likelihood of post-operative bleeding, swelling and infection [42]. When comparing our results with published studies, there was no significant increase in superficial or deep post-operative wound infections. The bikini incision follows Langer lines and been shown to facilitate wound healing in obese patients [34, 36]. In order to reduce the risk of infection the perineum was carefully isolated from the operative field using adhesive plastic drape with a “U” split.

### Leg length discrepancy

Leg length discrepancies were detected in two patients (0.23%) post-operatively in this report, similar to the 0.2% incidence reported by Leunig et al. [35]. The surgeon was aware of the discrepancy intraoperatively where 1 patient had a short leg after contralateral THR and the other patient had a dysplastic acetabulum. In our series, both legs were draped separately in all cases, enabling an accurate leg length check during the procedure.

### Iliopsoas tendinitis

All three cases of iliopsoas tendinitis in this report were treated initially with physiotherapy. One of them eventually underwent arthroscopic release resulting in complete relief of symptoms. Impingement of iliopsoas has been reported following DAA [13]. Preserving the anterio-medial capsule and avoiding oversizing of the cup could reduce the incidence of tendonitis.

### Femoral nerve injury

Femoral nerve injury, although infrequent, has been reported in DAA THR surgery. Cadaveric studies have highlighted that the position of the acetabular retractor tip may be as close as 2.8 mm to the nerve during DAA and anterior retractors can inadvertently stretch the femoral nerve [15, 51, 53]. In this study, the lead surgeon placed the retractors himself to avoid inadvertent stretching of the nerve, with the medial retractor placed at 7–8 o’clock position for preparation of a left acetabulum. We did not come across any case of femoral nerve injury in our series. In addition, the pneumatic broaching system minimises the time the operative leg is placed in extension, adduction and external rotation thereby minimising stretching of the femoral nerve [27].

#### Heterotrophic ossification

In addition to carefully protecting the abductors and minimizing soft tissue trauma, the use of LIA mixture with ketorolac and routine use of aspirin post-operatively may have helped prevent heterotrophic ossification (HO) as no cases were found to have HO until the most recent follow-up [8, 44]. Lower rates of HO has been reported in DAA when compared to the posterior approach [41].

### Other complications (DVT, trochanteric bursitis)

The incidence of deep vein thrombosis (DVT) and trochanteric bursitis following DAA THR varies widely in reported literature. Three patients (0.35%) reported a DVT post-operatively in this study compared to the 1.5% reported by Hallert et al. [20]. Trochanteric bursitis was diagnosed in eight patients (1.1%) in this series. In contrast, Shemesh et al. reported a higher 6.1% incidence of trochanteric bursitis in their series [48]. We recommend restoring the natural hip offset and performing an adhesiolysis using a finger sweep over the greater trochanter to reduce the risk of bursitis.

All cases in this study were elective, privately insured patients who were motivated in regards to early recovery and discharge. Limitations of this study include the operative surgeon’s fellowship training in DAA prior to commencement of the study, which likely impacted the learning curve and associated results. As this study was undertaken by a single surgeon in the private healthcare system, it was not possible to randomise and perform other surgical approaches as a comparison, where patients requested a DAA THR at initial consult. Our follow up is not long term, which may not address late failures resulting from this technique.

Despite recent systematic reviews demonstrating no significant advantages of DAA beyond six weeks and no significant difference in complication rates between DAA and posterior approach THR, joint registries highlight increasing adoption of DAA for THR [24, 26, 37]. Although DAA and particularly a bikini incision DAA is technically demanding, with appropriate training and precautions this approach may be safely performed without increased risk to the patient [12, 22, 43].

## Conclusion

We conclude that the bikini incision DAA may be used to perform THA with minimal complications on a standard orthopaedic operating table. Further large multicentre studies by suitably trained surgeons are encouraged.

## Data Availability

Data pertaining to all patients are stored in clinical records at Ozorthopaedics, Malvern, Melbourne and multiple hospitals in Melbourne, Australia. No identifiable patient data is present in the manuscript.

## Abbreviations

DAA: Direct anterior approach
THA: Total hip arthroplasty
HHS: Harris Hip scores
TFL: Tensor fascia latae
LFCN: Lateral femoral cutaneous nerve
Womac scores: The Western Ontario and McMaster Universities Osteoarthritis Index
